# Utility of Echo Planar Imaging With Compressed Sensing-Sensitivity Encoding (EPICS) for the Evaluation of the Head and Neck Region

**DOI:** 10.7759/cureus.54203

**Published:** 2024-02-14

**Authors:** Yuya Hirano, Noriyuki Fujima, Kinya Ishizaka, Takuya Aoike, Junichi Nakagawa, Masami Yoneyama, Kohsuke Kudo

**Affiliations:** 1 Department of Radiological Technology, Hokkaido University Hospital, Sapporo, JPN; 2 Department of Diagnostic and Interventional Radiology, Hokkaido University Hospital, Sapporo, JPN; 3 Department of Clinical Sciences, Philips Japan, Tokyo, JPN; 4 Department of Diagnostic Imaging, Hokkaido University Graduate School of Medicine, Sapporo, JPN

**Keywords:** diffusion-weighted imaging (dwi), echo-planar imaging (epi), head and neck, magnetic resonance imaging, compressed sensing

## Abstract

Purpose

This study aimed to compare the image quality between echo planar imaging (EPI) with compressed sensing-sensitivity encoding (EPICS)-based diffusion-weighted imaging (DWI) and conventional parallel imaging (PI)-based DWI of the head and neck.

Materials and methods

Ten healthy volunteers participated in this study. EPICS-DWI was acquired based on an axial spin-echo EPI sequence with EPICS acceleration factors of 2, 3, and 4, respectively. Conventional PI-DWI was acquired using the same acceleration factors (i.e., 2, 3, and 4). Quantitative assessment was performed by measuring the signal-to-noise ratio (SNR) and apparent diffusion coefficient (ADC) in a circular region of interest (ROI) on the parotid and submandibular glands. For qualitative evaluation, a three-point visual grading system was used to assess the (1) overall image quality and (2) degree of image distortion.

Results

In the quantitative assessment, the SNR of the parotid gland in EPICS-DWI was significantly higher than that of PI-DWI in acceleration factors of 3 and 4 (p<0.05). In a comparison of ADC values, significant differences were not observed between EPICS-DWI and PI-DWI. In the qualitative assessment, the overall image quality of EPICS-DWI was significantly higher than that of PI-DWI for acceleration factors 3 and 4 (p<0.05). The degree of image distortion was significantly larger in EPICS-DWI with an acceleration factor of 2 than that of 3 or 4 (p<0.01, respectively).

Conclusion

Under the appropriate parameter setting, EPICS-DWI demonstrated higher SNR and better overall image quality for head and neck imaging than PI-DWI, without increasing image distortion.

## Introduction

Diffusion-weighted imaging (DWI) is one of the important techniques in magnetic resonance imaging (MRI) that can obtain microstructural characteristics by measuring water diffusivity in various tissues, including cancer. The utility of DWI for the evaluation of head and neck lesions, including their detection, differentiation as malignant or benign, and prognostication, has been widely reported [1-7].

However, MRI acquisition of the head and neck region is challenging because of B0 and B1 field inhomogeneity due to the complicated structures, presence of air, and effects of metallic artifacts; this results in low image quality with severe image distortion [8]. Single-shot spin-echo echo planar imaging (EPI) with the use of parallel imaging (PI) is the conventional imaging sequence for DWI, with the main advantage being its fast readout. To prevent image distortion in this sequence, a lower-number EPI factor is essential, which is obtained by using a higher parallel-imaging acceleration factor. However, a higher-number acceleration factor may result in bulk noise and a lower signal-to-noise ratio (SNR), especially in regions with complex geometric factors [9]. Turbo spin echo (TSE)-based DWI is another technique in which image distortion is largely restricted due to the spin echo-based readout scheme [10]. However, this technique requires a very long scanning time (typically four to six minutes) compared to conventional EPI-based DWI (typically one to two minutes) [11]. In addition, the image SNR is not necessarily high because of the combination of the long echo space and a large number of TSE factors [12]. Therefore, another technique for head and neck DWI imaging is needed.

Recently, the DWI technique of EPI with compressed sensing-sensitivity encoding (SENSE), named "EPICS," was introduced [13-16]. CS-SENSE is an effective combination technique of compressed sensing and PI [17,18]. Specifically, it is an image reconstruction technique using the undersampling of k-space data with sparse image signals and a repeated denoising cycle based on an iterative L1-regularized denoising filter (i.e., wavelet transformation). This technique enables a marked reduction of sampling data in k-space without much decrease in the SNR [19]. Although CS-SENSE has not yet been applied in the EPI readout, in recent years, this technique has become available under the name of EPICS, as mentioned above. Several preliminary investigations were conducted targeting various parts of the human body, such as the brain, abdomen, and prostate, as well as the head and neck [15,16,20-22].

This study aimed to investigate the utility of EPICS-DWI for the evaluation of the head and neck region. For this purpose, we performed quantitative and qualitative evaluations to assess image quality and the degree of image distortion for the comparison between EPICS-DWI and PI-DWI in various reduction factors to determine the appropriate reduction factor for the use of EPICS-DWI.

## Materials and methods

Subjects

The study was approved by the Institutional Review Board of Hokkaido University Hospital (approval number: 017-0455), and written informed consent was obtained from all participants. Ten healthy volunteers (10 men, 30.5±2.9 years, age range 26-34) participated in this study.

MRI scanning

All MRI scanning was performed using a 3-Tesla MR unit (Ingenia Elition; Philips Healthcare, Best, the Netherlands) with a 16-channel head-neck coil. EPICS-DWI was acquired based on the axial spin-echo EPI sequence with EPICS acceleration factors of 2, 3, and 4, respectively, using the following imaging parameters: TR 4000 ms, TE 66-53 ms, slice thickness 5 mm, FOV 240×240 mm, acquisition voxel size 1.8×1.8 mm (reconstruction voxel seize 0.9×0.9 mm), number of EPI factors 61-31, scan time 72 seconds, b-values 0, and 1000 mm^2^/sec. In addition, conventional PI-based DWI (PI-DWI) was acquired with parallel-imaging acceleration factors (i.e., sensitivity encoding factor; SENSE factor) of 2, 3, and 4, respectively, the same imaging parameters used as in the EPICS-DWI protocol described above. For both PI-DWI and EPICS-DWI, the acquisition was performed at the same scanning time (=72 seconds) for all acceleration factors. The details of the scanning protocol are presented in Table 1.

**Table 1:** Scan parameters of EPICS-DWI and PI-DWI TR: repetition time, TE: echo time, EPI: echo planar imaging

Acceleration factor	2	3	4
TR (ms)	4000	4000	4000
TE (ms)	66	57	53
Acquisition matrix size (mm)	1.8×1.8	1.8×1.8	1.8×1.8
Reconstruction matrix size (mm)	0.9×0.9	0.9×0.9	0.9×0.9
Slice thickness (mm)	5	5	5
EPI factor	61	41	31
Number of slices	19	19	19
b-value (mm^2^/sec)	0, 1000	0, 1000	0, 1000
Scan time (sec)	72	72	72

The scanning range was set to include the entire neck so that the parotid gland was placed at the center of the slices. Fat-suppressed axial T2-weighted imaging (Fs-T2WI) was also acquired as a reference with the following parameters: TR 4197 ms, TE 90 ms, slice thickness 5 mm, FOV 240×240 mm, and acquisition voxel 1.8×1.8 mm (reconstruction voxel 0.9×0.9 mm).

Data analysis

Quantitative Assessment

Quantitative assessments were performed by measuring the SNR and apparent diffusion coefficient (ADC). In the SNR measurement, a 10-mm circular region of interest (ROI) was placed on the parotid gland and submandibular gland in both EPICS-DWI and PI-DWI with a b-value of 1000 mm^2^/sec. This procedure was performed by referring to b0 images and Fs-T2WI to avoid major vascular structures being included in the ROI. All procedures in ROI measurement were performed using Image J software (National Institutes of Health, software version 1.52d). The quantitative analysis was conducted by an experienced radiological technologist. The SNR measurement of the parotid gland in the circular ROI was performed with the following equation:

SNR = (μ_ROI_) / (SD_ROI_) (1)

where μ_ROI_ is the mean signal intensity in the ROI placed on each target, and the SD_ROI_ is the standard deviation of the signal intensity in the ROI. In the ADC calculation, ROIs placed on EPICS- and PI-DWI with b-values of 1000 mm^2^/sec for SNR measurement were copied onto corresponding b0 images. Subsequently, the mean signal intensity in ROIs was used for the ADC value calculation with the following equation:

(μ_b=1000_) / (μ_b=0_) = exp(−1000*ADC) (2)

where μ_b=1000_ and μ_b=0_ were the mean signal intensities in the ROI placed on the b=1000 mm^2^/sec and b=0 mm^2^/sec images, respectively.

Qualitative Assessment

A qualitative visual assessment was performed to evaluate the overall image quality and the degree of the image distortion. An evaluation was independently performed by two radiologists with 15 and six years of experience in head and neck imaging. The radiologists reviewed all slices of EPICS-DWI and PI-DWI of b=1000 images with the DICOM viewer in a blinded fashion: all image information such as a sequence detail (EPICS-DWI or PI-DWI) and acceleration factor (i.e., 2, 3, or 4) was withheld from the rater. Overall image quality was evaluated based on the visibility of the normal structure and the presence of image noise with a three-point grading system (1, poor; 2, moderate; 3, good). The degree of distortion was also evaluated based on a three-point scale (1, moderate/severe distortion; 2, slight distortion; 3, almost no distortion); in the current study, both moderate and severe were treated with the same score, considering that both could have a considerable impact on diagnostic accuracy.

Statistical analysis

In quantitative analysis, the values of SNR and ADC for each acceleration factor (2, 3, and 4) were compared between EPICS-DWI and PI-DWI using the Wilcoxon signed rank test. In the qualitative analysis, the score of the overall image quality for each acceleration factor was compared between EPICS-DWI and PI-DWI, also using the Wilcoxon signed rank test. In addition, the score of the degree of image distortion in EPICS-DWI was compared among the three acceleration factors (2, 3, and 4) using the Kruskal-Wallis test. The agreement between the two radiologists in the visual evaluation was measured using Kappa statistics (0.00-0.20, poor; 0.21-0.40, fair; 0.41-0.60, moderate; 0.61-0.80, good; and 0.81-1.00, excellent). P-values <0.05 were accepted as significant. JMP Pro 16.0.0 (SAS Institute, Cary, NC, USA) was used for all statistical analyses.

## Results

All scanning was successfully performed without any complications. All images were deemed acceptable for the quantitative and qualitative evaluations.

In the quantitative assessment, the SNR value of the parotid gland in EPICS-DWI with an acceleration factor of 3 (right: 13.3±3.0, left: 13.1±4.3) was significantly higher than that in PI-DWI with an acceleration factor of 3 (right: 9.8±1.6, left: 9.3±2.6) (p<0.05), in both the right and left sides, respectively. The SNR value of the bilateral parotid glands in EPICS-DWI with an acceleration factor of 4 (right: 12.8±3.2, left: 13.0±6.0) was also significantly higher than that in PI-DWI with an acceleration factor of 4 (right: 8.0±1.8, left: 8.5±2.3) (p<0.05), respectively. In contrast, the SNR value of the bilateral submandibular glands in EPICS-DWI with an acceleration factor of 3 and 4 tended to be higher than PI-DWI. However, statistical significance was not observed. Details of the SNR assessment are presented in Table 2.

**Table 2:** SNR values in EPICS-DWI and PI-DWI Data are means ± standard deviations. * indicates statistical significance. SNR: signal-to-noise ratio, EPICS, echo planar imaging with compressed sensing-sensitivity encoding, DWI: diffusion-weighted imaging, PI: parallel imaging

	EPICS-DWI	PI-DWI	p-value	EPICS-DWI	PI-DWI	p-value	EPICS-DWI	PI-DWI	p-value
	Acceleration factor 2			Acceleration factor 3			Acceleration factor 4		
Rt. parotid gland	13.9±3.1	12.4±2.9	0.57	13.3±3.0	9.8±1.6	0.003*	12.8±3.2	8.0±1.8	0.004*
Lt. parotid gland	13.8±4.7	10.7±2.0	0.12	13.1±4.3	9.3±2.6	0.01*	13.0±6.0	8.5±2.3	0.03*
Rt. subman-dibular gland	8.1±2.8	8.1±2.8	0.85	8.9±3.9	6.6±2.1	0.21	8.5±2.6	6.7±2.2	0.3
Lt. subman-dibular gland	8.1±2.9	7.1±2.0	0.57	7.3±2.8	6.4±1.8	0.47	6.6±1.6	5.8±1.7	0.34

In a comparison of ADC values, the ADC values in the EPICS-DWI only with acceleration factors 4 showed significantly higher values than the PI-DWI in the right parotid gland and bilateral submandibular gland, respectively (p<0.05). Details of the ADC assessment are presented in Table 3.

**Table 3:** ADC values in EPICS-DWI and PI-DWI Data are means ± standard deviations. * indicates statistical significance. ADC: apparent diffusion coefficient, EPICS: echo planar imaging with compressed sensing-sensitivity encoding, DWI: diffusion-weighted imaging, PI: parallel imaging

	EPICS-DWI	PI-DWI	p-value	EPICS-DWI	PI-DWI	p-value	EPICS-DWI	PI-DWI	p-value
	Acceleration factor 2			Acceleration factor 3			Acceleration factor 4		
Rt. parotid gland	0.81±0.16	0.75±0.18	0.51	0.82±0.13	0.71±0.16	0.17	0.89±0.15	0.70±0.14	0.03*
Lt. parotid gland	0.78±0.14	0.73±0.13	0.64	0.75±0.13	0.72±0.15	0.79	0.79±0.13	0.69±0.11	0.15
Rt. subman-dibular gland	1.20±0.09	1.15±0.11	0.47	1.17±0.07	1.16±0.11	0.84	1.35±0.18	0.99±0.11	0.0007*
Lt. subman-dibular gland	1.11±0.24	0.98±0.17	0.16	1.07±0.24	0.99±0.18	0.4	1.15±0.27	0.88±0.19	0.02*

The Kappa scores for the qualitative visual assessment scales between the two readers for both overall image quality and the degree of the image distortion are excellent (overall image quality: 0.713, 95% CI: 0.559-0.868, and degree of the image distortion: 0.789, 95% CI: 0.563-1.015). In the qualitative visual assessment, the overall image quality of EPICS-DWI with an acceleration factor of 3 was significantly higher than that of PI-DWI in both readers 1 and 2 (p<0.01). Additionally, the overall image quality in EPICS-DWI with an acceleration factor of 4 was also significantly higher than that in PI-DWI in both readers 1 and 2 (p<0.05). Details of overall image quality are presented in Table 4.

**Table 4:** Results of visual assessment; overall image quality in EPICS-DWI and PI-DWI Data are means ± standard deviations. * indicates statistical significance. ADC: apparent diffusion coefficient, EPICS: echo planar imaging with compressed sensing-sensitivity encoding, DWI: diffusion-weighted imaging, PI: parallel imaging

	Reader 1			Reader 2		
	EPICS-DWI	PI-DWI	p-value	EPICS-DWI	PI-DWI	p-value
Acceleration factor 2	3±0	2.7±0.5	0.07	2.9±0.3	2.5±0.5	0.06
Acceleration factor 3	2.7±0.5	2.0±0	0.001*	2.8±0.4	1.9±0.3	0.0005*
Acceleration factor 4	2.1±0.3	1.5±0.7	0.02*	2.3±0.5	1.4±0.7	0.005*

**Figure 1:**
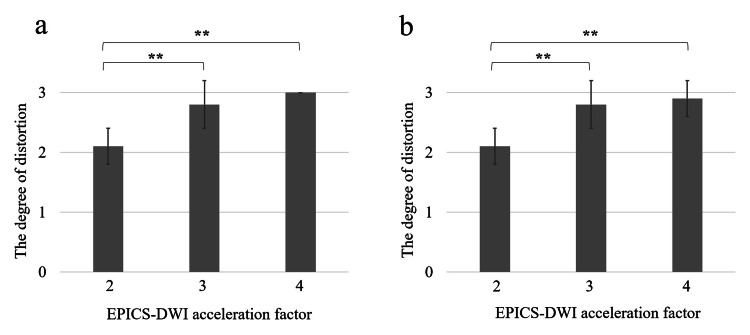
Degree of the image distortion in EPICS-DWI The degree of image distortion evaluated by two radiologists was presented respectively (a and b). In both radiologists, the degree of image distortion was significantly larger in EPICS-DWI with an acceleration factor of 2 than that of 3 and 4 (* statistical significance), whereas there was no significant difference between acceleration factors of 3 and 4

The degree of image distortion was significantly larger in EPICS-DWI at an acceleration factor of 2 than that at factors of 3 and 4 (p<0.01, respectively). Details of the degree of image distortion are presented in Figure 1.

**Figure 2:**
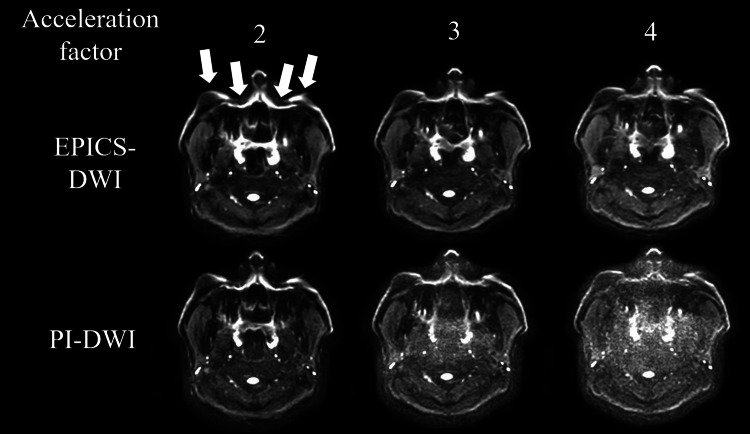
Representative case of both EPICS-DWI and PI-DWI Axial EPICS-DWIs and PI-DWIs in a 33-year-old male were presented. Particularly, EPICS-DWI with an acceleration factor of 3 demonstrated both less image noise and less image distortion. Compared to this image, PI-DWI and EPICS-DWI with an acceleration factor of 2 maintained a certain degree of image distortion (arrows). In addition, moderate or severe image noise was observed in PI-DWI with an acceleration factor of 3 and 4 and EPICS-DWI with an acceleration factor of 4

A representative case of EPICS-DWI and PI-DWI among all acceleration factors is presented in Figure 2.

## Discussion

In the current study, we demonstrated that with appropriate image parameter settings, EPICS-DWI could perform with higher overall image quality and less image distortion than PI-DWI for imaging the head and neck region. We also demonstrated a higher regional SNR on the parotid gland with EPICS-DWI than PI-DWI, although the submandibular gland did not show a significant difference in SNR. EPICS-DWI has the potential to play an important role as a new conventional DWI sequence with improved image quality for evaluation of the head and neck region.

In recent decades, conventional PI-DWI with single-shot spin-echo EPI has been frequently used for the evaluation of the head and neck [23]. In this sequence, a high PI acceleration factor to allow for a lower EPI factor has been required to reduce the image distortion appearing along the phase encode direction. This is particularly true in the evaluation of the head and neck, where B0 and B1 field inhomogeneity is frequently present [24]. However, an excessive acceleration factor in PI always results in image noise, especially in pixels with high geometry factors; this causes lower-SNR images [25]. From this point of view, conventional spin-echo EPI-based DWI with a PI technique makes it difficult to obtain images with both a high SNR and adequately limited image distortion [9]. In contrast, the CS-SENSE algorithm in EPICS-DWI more effectively decreases the image noise even in pixels with high geometry factors by adopting a denoising cycle as a post-processing feature [14,15]. As a result, this technique enables an increase in the acceleration factor to reduce image distortion while maintaining the image SNR using CS-SENSE post-processing.

In the current study, an EPICS acceleration factor of 3 and 4 demonstrated higher SNR compared to PI-DWI. In contrast, most of the participants were scored as good for overall image quality assessment in the EPICS-DWI with an acceleration factor of 3, while several participants were classified as moderate in the EPICS-DWI with an acceleration factor of 4. At an acceleration factor of 4, excessive image noise is likely in voxels with high geometry factors, such that even the CS-SENSE denoising cycle cannot sufficiently reduce the image noise. In comparison among the acceleration factors of 2-4 from the viewpoint of image distortion, a certain degree of image distortion was observed with the acceleration factor of 2. At an acceleration factor of 2, the EPI factor becomes too large and causes marked image distortion in the phase encoding direction, particularly in regions with remarkable B0 or B1 field inhomogeneity like the head and neck. Considering these viewpoints, the appropriate setting of the EPICS-DWI acceleration factor for head and neck DWI might be 3 under the imaging parameter setting used in the present study. Previously, Yoshida et al. investigated the utility of EPICS-DWI and provided improved image quality compared to conventional PI-DWI with an acceleration factor of 4 [16]. This result was consistent with the current study. However, they assessed the EPICS-DWI with an acceleration of 4 only. The current study provided the image quality and the degree of distortion with acceleration factors of 2-4. Therefore, we speculate that the information revealed in the current study is useful for optimal use, depending on the purpose of the individual MR examination.

In the ADC assessment, the value of ADC in the EPICS-DWI with acceleration factors 4 showed significant differences in the right parotid gland and bilateral submandibular gland, although no significant difference was observed in other acceleration factors. The reason for this was still unclear; however, we speculated the denoising effect in EPICS-DWI might affect the tissue signal intensity on images, and thus signal differences between EPICS- and PI-DWI resulted in the differences in ADC values. The degree of difference in ADC between EPICS-DWI and PI-DWI for acceleration factors 2 and 3 was very small, around 0.1 x 10-3 mm^2^/s. With such a small difference, the influence of the calculated values of ADC obtained from EPICS-DWI for each acceleration factor 2 and 3 on clinical use is mostly considered negligible when compared to the ADC obtained from PI-DWI. If we used the EPICS-DWI with an acceleration factor of 4, the interpretation of the ADC value should be carefully treated.

The current study has several limitations. First, the number of subjects was small. Although the quantitative SNR and qualitative overall image quality in EPICS-DWI showed a statistically significant improvement compared to PI-DWI, the results of the current study should be regarded as preliminary. Second, we evaluated normal, healthy subjects only; actual lesions in the head and neck were not evaluated. Third, in recent years, there has been remarkable progress in deep learning-based image reconstruction, including head and neck imaging [26,27]. A comparison of image quality between EPICS and deep learning-based image reconstruction in DWI may be valuable. Further studies will be needed to address these limitations.

## Conclusions

The EPICS-DWI shows less deterioration in image quality with an increasing acceleration factor than the PI-DWI. This technique permits a more substantial increment in the acceleration factor for acquiring head and neck DWI while maintaining better image quality and minimizing geometric distortion. In the current study, employing EPICS with an acceleration factor of 2.0 led to a noticeable degree of image distortion. Conversely, an acceleration factor of 4.0 introduced a minor but persistent image noise that remained unresolved even with the application of the EPICS technique. It is suggested that an acceleration factor of approximately 3.0 may be optimal for acquiring head and neck DWI at a standard spatial resolution and scanning time. This technique has the potential to provide better diagnostic performance in the field of head and neck radiology.
